# Nickel-containing nano-sized islands grown on Ge(111)-c(2 × 8) and Ag/Ge(111)-(√3 × √3) surfaces

**DOI:** 10.1186/1556-276X-8-416

**Published:** 2013-10-08

**Authors:** Tsu-Yi Fu, Agnieszka Tomaszewska, Xiao-Lan Huang, Jhen-Hao Li, Po-I Hsieh, Ming-Kuan Jhou

**Affiliations:** 1 Department of Physics, National Taiwan Normal University, 88, Sec. 4 Ting-Chou Rd., Taipei 116, Taiwan; 2 Institute of Physics, Jan Długosz University, Armii Krajowej Ave. 13/15, Częstochowa 42-200, Poland

**Keywords:** Ni, Ag, Ge(111), STM

## Abstract

The formation of nano-islands on both a Ge(111)-c(2 × 8) surface and an Ag/Ge(111)-(√3 × √3) surface evaporated with 0.1 ML Ni was investigated by scanning tunneling microscopy (STM). We have noticed that at temperatures lower than 670 K, the reaction between Ni and the individual substrate surfaces proceeds to form different structures: flat-topped islands with a 2√7 × 2√7 or a 3 × 3 reconstruction on the Ni/Ge(111)-c(2 × 8) surface vs. islands with a 7 × 7 reconstruction on the Ni/Ag/Ge(111)-(√3 × √3) surface. From this we have inferred that within a temperature range between room temperature and 670 K, the intermediate Ag layer retards mixing between Ni and Ge atoms. As a result, the grown islands are composed of pure Ni atoms. Within a temperature range from 670 to 770 K, most islands produced on the Ag/Ge(111)-(√3 × √3) surface are identical with those formed on the Ni/Ge(111)-c(2 × 8) surface, suggesting that above 670 K, Ni atoms are likely to bind with Ge atoms. However, an essential difference between STM images of the surfaces under study exists in the appearance of large elongated islands on the Ni/Ag/Ge(111)-(√3 × √3) surface. The formation of the latter is explained in terms of a difference in energy for Ni diffusion on the Ge(111)-c(2 × 8) and Ag/Ge(111)-(√3 × √3) surfaces.

## Background

An extraordinary interest in the growth of thin metal layers on a semiconductor substrate is driven by the application of metal/semiconductor interfaces as ohmic contacts for electronic devices. In particular, the reaction of 3D transition metals (TMs) (such as Co, Ni, Fe) with different Si and Ge surfaces has attracted a great deal of attention on account of the importance of the resulting compounds to magnetic storage media [1-10]. In order to achieve a better understanding of the complex phenomena which occur during silicide or germanide formation, detailed studies of the very early stages of a film preparation should be performed. Particularly important are studies directed toward characterization of the morphology of the interface formed by deposition of small amounts of TMs onto the semiconductor surface because there exists a correlation between surface morphology and electronic, optical, and magnetic properties of the surface. Introducing foreign metal atoms into the metal/semiconductor system opens a possibility to induce some significant changes in surface morphology which, in turn, translate into changes in the above-mentioned properties of the surface. For example, Tsay et al. have found that Co films grown on an Ag/Ge(111) surface exhibit magnetic properties, which contrast with the non-ferromagnetic properties of a Co/Ge(111) surface [11]. This finding was interpreted in terms of buffering properties of the intermediate Ag layer, which prevent the deposited Co atoms from germanide formation. The remarkable properties of the Co/Ag/Ge(111) surface system inspired the work in our laboratory, where, in the last several years, attention was paid to the characterization of the early stages of Co nucleation on the Ag/Ge(111) surface by means of scanning tunneling microscopy (STM) [12-14]. By comparing the method of the Ag/Ge(111) surface fabrication used by Tsay et al. with the Ag/Ge(111) surface diagram [15], we ascribed the buffering properties to the √3 × √3 phase and explained them in the light of the existing structural models of the latter [16,17]. Briefly, in the √3 × √3 structure, both the Ag atoms and the outermost Ge atoms are arranged in a triangular configuration. The formation of a Ge triangle satisfies two of three surface dangling bonds, and the remaining bond is saturated with an Ag atom. Therefore, the deposited Co atoms cannot readily combine with Ge(111) surface atoms, and the surface remains passive toward the adsorbate. We have also found that early stages of Co film formation on the Ag/Ge(111)-√3 × √3 surface are determined by the formation of islands with either √13 × √13 or 2 × 2 reconstruction. Interestingly, a recent STM study of Co growth on a bare Ge(111)-c(2 × 8) surface (the native reconstruction of the Ge(111) surface) has revealed the formation of islands with the same reconstruction patterns [10].

This finding has motivated us to perform a comparative study of the early stages of Ni nucleation on the Ge(111)-c(2 × 8) and Ag/Ge(111)-√3 × √3 surfaces and reinvestigate the concept of the buffering properties of the latter surface. From literature overview it seems that the interactions at a Ni/Ge(111) interface considerably differ in nature from those on the Ag/Ge(111) interface. The growth of Ni on the Ge(111) surface has been described as a complicated case in which the formation of surface compounds occurs [4]. Even at room temperature (RT), the mobility of Ni and Ge atoms is not negligible, and the Ni atoms in the deposited layer are being replaced by Ge atoms [5]. In contrast, at the Ag/Ge interface, the intermixing between the species is limited [18]. Depending on the coverage, the annealed Ag/Ge interface develops three different reconstruction patterns: 4 × 4, 3 × 1, and √3 × √3 [19]. The Ag/Ge(111)-√3 × √3 surface is formed when the Ag coverage is around 1 ML. In the surface, metal atoms are strongly bound to the semiconductor substrate surface and they are therefore hard to move from their sites.

In our study we restrict attention to small Ni coverage in order to follow the formation of nano-sized objects. We hope that our findings will be useful for controlling the nano-island growth on the surface.

## Methods

Experiments were performed with a commercial ultrahigh-vacuum, variable-temperature scanning tunneling microscope (UHV-VT STM, Omicron, Taunusstein, Germany). Prior to deposition, p-type Ge(111) wafers (1 to 10-Ω cm resistivity, 0.5-mm thickness) were cleaned *in situ* at a base pressure of 2 × 10^-8^ Pa by repeated cycles of Ar^+^ bombardment (1.0 keV, 10° to 90° incidence angle), followed by annealing at 923 K for 1 to 2 h and then cooling at a rate of around 1 K/min. The Ag/Ge(111)-√3 × √3 surface was prepared by exposing the Ge(111)-c(2 × 8) surface, kept at RT, to an Ag beam from a K-cell dispenser for 90 min, followed by annealing at approximately 773 K. As a result of this treatment, approximately 1 ML Ag remains on the surface, which is enough to produce the wanted √3 × √3 phase. Ni atoms from an e-beam evaporator were deposited at a fixed rate of 0.1 ML/min onto either the clean Ge(111)-c(2 × 8) or the Ag/Ge(111)-√3 × √3 surface, dependently on the desirable final adsorption system. During deposition, the substrates were kept at RT and the pressure did not exceed 2 × 10^-7^ Pa. For growth promotion, the surfaces with deposited materials were post-annealed within a range of 373 to 873 K for 30 min. From our experience, annealing for at least 30 min is necessary to obtain the thermal equilibrium surface. The sample temperature below 450 K was measured using a silicon diode, whereas that above 873 K was read from an optical pyrometer. In addition, K-type thermocouple was used to measure the temperature within the whole applied range. All STM images presented in this paper were acquired at room temperature using KOH-etched W tips.

## Results and discussion

**Figure 1:**
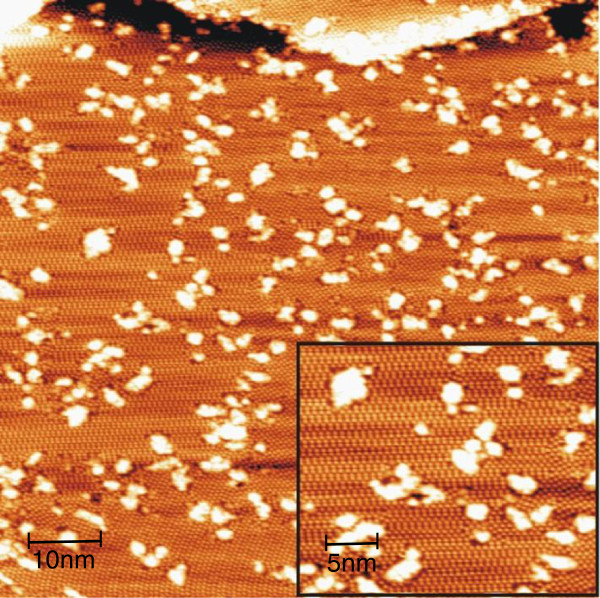
**Empty-state STM image showing the formation of clusters after Ni deposition onto Ge(111)-c(2 × 8) surface at RT.** The initial Ni coverage is approximately 0.1 ML. The image size and bias voltage are 80 × 80 nm^2^ and 1.5 V, respectively. Inset: small-scale (30 × 25 nm^2^) image zoomed from the large area showing that clusters have a tendency to accumulate at boundaries between the different c(2 × 8) domains.

The Ge(111) surface, prepared under the conditions described in the previous section, shows the tendency to display the c(2 × 8) domains of different orientations in coexistence with small domains of local 2 × 2 and c(4 × 8) symmetry. After deposition of 0.1 ML Ni onto the surface (Figure 1), we can observe the formation of brightly imaged clusters. The clusters accumulate predominantly at the boundaries between either the different domains which exist on the surface or the different c(2 × 8) orientations (see inset in Figure 1). The abundance of the clusters is also seen at the edge separating the terraces, implying that the RT mobility of Ni is not negligible. The inner parts of domains are covered by clusters at a smaller degree. A closer inspection reveals that most clusters are surrounded by dark holes in the substrate which indicates that even at RT, metallic adsorbate reacts with Ge. The formation of Ni-induced structural defects in semiconductor surfaces has been widely reported in the literature of the subject, e.g., [20].

**Figure 2:**
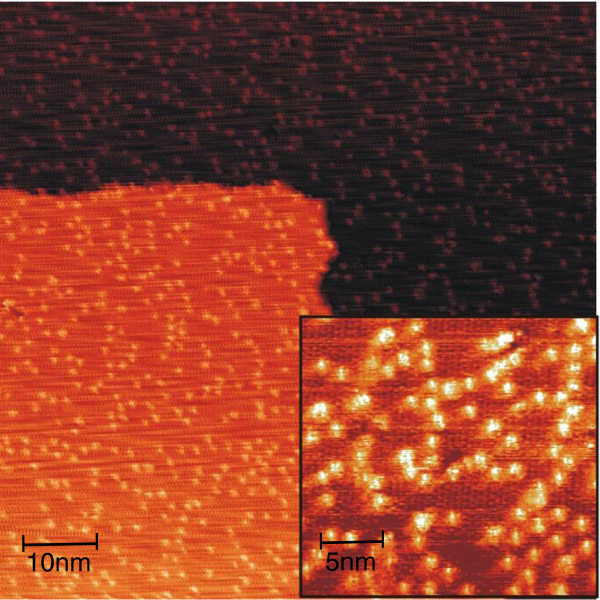
**Filled-state STM image taken after deposition of 0.1 ML Ni onto Ag/Ge(111)-√3 × √3 surface at RT.** The image size is 80 × 80 nm^2^, and the bias voltage is -1.6 V. Inset: small-scale (24 × 22 nm^2^) image showing that clusters are randomly distributed on the surface.

Figure 2 shows the Ag/Ge(111)-√3 × √3 surface with 0.1 ML Ni deposited at RT. Here, clusters seem to be randomly distributed without concentrating at the terrace edges, which indicates that the surface diffusion of the species at RT is suppressed. In the area between the clusters, a defect-free √3 × √3 structure is clearly resolved (see inset in Figure 2) which suggests that there is no chemical reaction between the deposit and the surface. Therefore, we argue that the clusters are composed of pure Ni atoms rather than Ni-Ge compounds.

**Figure 3:**
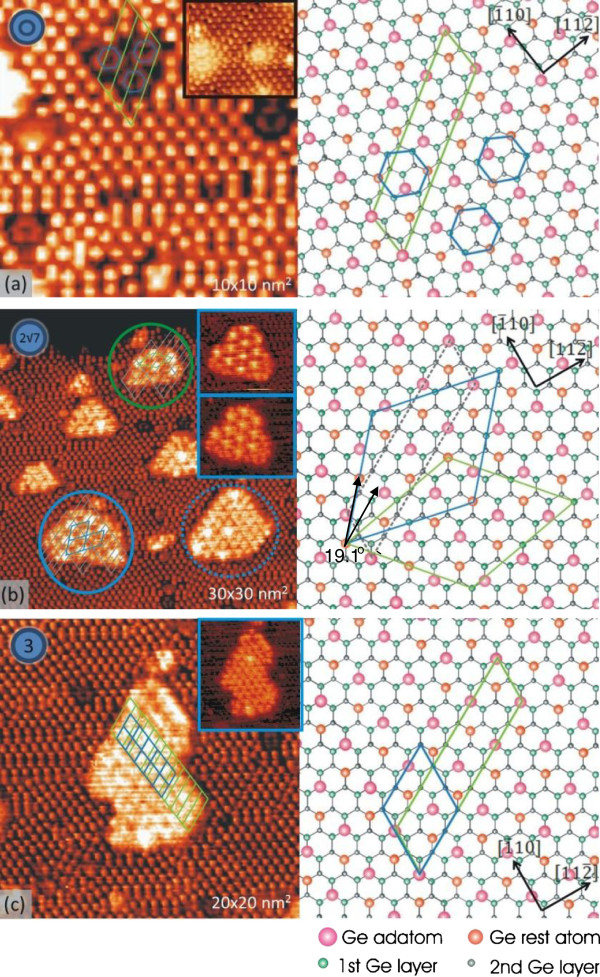
**STM images showing Ni-induced structures on Ge(111)-c(2 × 8) surface. (a)** Ring-like defects in single and trimer configurations. Inset: 7 × 7 nm^2^ filled-state image taken at a sample bias of -0.6 V, showing ring-like defects. **(b)** 2√7 × 2√7 islands are enclosed by solid circles, whereas the 3 × 3 island is enclosed by a dotted circle. Insets: 12 × 10 nm^2^ images of the same 2√7 × 2√7 island taken at a positive (upper inset) and a negative (lower inset) bias voltage. **(c)** Empty-state image of a magnified 3 × 3 island. Inset: 13 × 15 nm^2^ filled-state image of the same island. Image size is indicated in each image. The notations in left upper corners represent the specified structures. Corresponding schematic diagrams against a background of the Ge(111)-c(2 × 8) structure are shown in right half parts.

**Figure 4:**
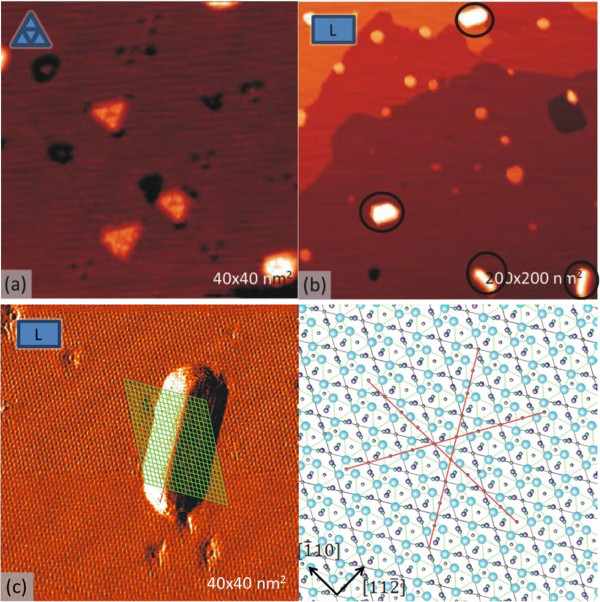
**Empty-state STM images showing Ni-containing structures on Ag/Ge (111)-√3 × √3 surface. (a)** Triple-hole defects which appear after annealing between 470 and 570 K. **(b)** Long islands (enclosed by circles) which appear after annealing above 670 K. **(c)** Zoomed long island with a corresponding schematic diagram of Ag/Ge(111)-√3 × √3 surfaces in which three directions for island growth are indicated.

**Figure 5:**
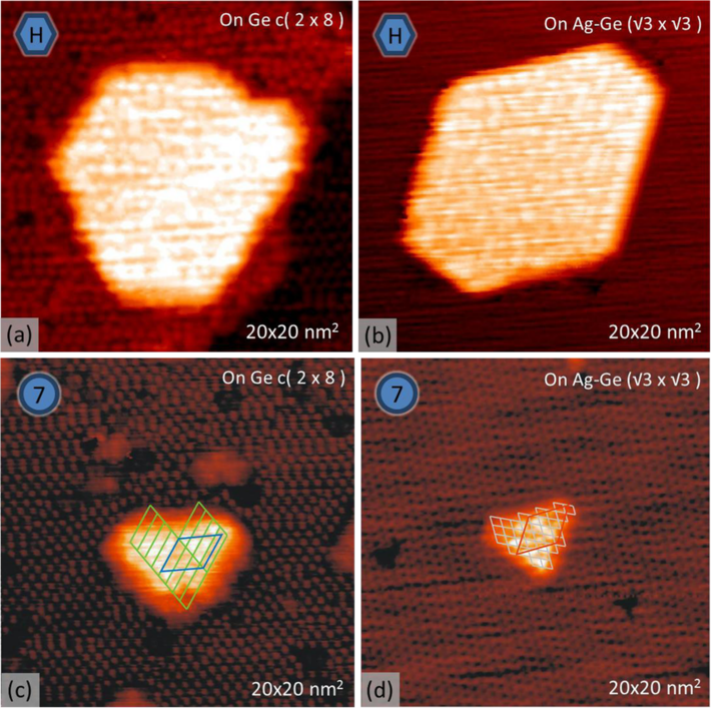
**Empty-state STM images showing Ni-containing structures. (a)** Hexagonal island on Ge(111)-c(2 × 8) surface. **(b)** Hexagonal island on Ag/Ge(111)-√3 × √3 surfaces. **(c)** 7 × 7 island on Ge(111)-c(2 × 8) surface. **(d)** 7 × 7 island on Ag/Ge(111)-√3 × √3 surfaces. The notations in left upper corners represent the specified structures.

Annealing the surfaces with deposited materials within the range from 470 to 770 K results in the appearance of a variety of objects. While most of them appear only on either Ni/Ge(111)-c(2 × 8) surface (Figure 3) or Ni/Ag/Ge(111)-√3 × √3 surface (Figure 4), some structures commonly form on both of them (Figure 5).

First, we focus on the structures typical of the Ni/Ge(111)-c(2 × 8) surface. They are presented in Figure 3 along with proposed schematics of the structural models. The models are drawn on a background of the Ge(111)-c(2 × 8) lattice. Figure 3a is a small-scale empty-state STM image showing ring-like defects. By analyzing a number of images, we have found that the structures emerge in single, dimer, or trimer configuration. In an attempt to explain the origin of the structures, we shall recall that ring-like clusters frequently develop after annealing the Si(111) surfaces containing trace amounts of Ni [1], Co [3], and Fe [6]. Depending on the adsorption system, the authors ascribed the rings to precursors to either metal-induced reconstruction of the substrate surface or metal-containing islands which grow on the substrate surface. The ring-like defects, however, were not reported on the Co/Ge(111)-c(2 × 8) surface [10].

By referring the STM image to the structural model of the Ge(111)-c(2 × 8) (Figure 3a), we notice that the rings are likely to represent missing Ge adatoms. In filled-state images, however, the rings are brighter in contrast to the substrate. This effect is particularly distinct for the sample bias -0.6 V at which no local density of states exists for the Ge(111)-c(2 × 8) surface (see inset in Figure 3a). This observation leads us to conclude that the ring-like defects are more likely to belong to Ni atoms sitting at Ge atom positions rather than represent missing adatoms.

Besides the ring-like defects, annealing the Ni/Ge(111)-c(2 × 8) surface produces flat-topped islands with atomically resolved corrugations, forming a 2√7 × 2√7 pattern (islands enclosed with solid circles in Figure 3b) and a 3 × 3 pattern (in Figure 3b, the island enclosed with a dotted circle). The islands typically have a height within the range from 0.15 to 0.2 nm and adopt approximately triangular, hexagonal, and trapezoidal shapes. However, a few islands are observed with irregular shapes. The islands with the 3 × 3 are observed at higher densities as compared to their counterparts. The distances between the islands and ring-like objects as well as their location on the surface are random. More detailed features of the different islands are shown in the insets in Figure 3b as well as in Figure 3c. We shall notice that both islands have empty-state images markedly different from the filled-state ones. This indicates that the islands have semiconducting properties rather than metallic. The unit cell of the 3 × 3 structure is aligned parallel to the c(2 × 8) unit cell (right part of Figure 3c) while that of the 2√7 × 2√7 structure is rotated by an angle of 19.1° to the c(2 × 8) unit cell, as illustrated in Figure 3b.

Figure 4 shows structures which grow on the annealed Ni/Ag/Ge(111)-√3 × √3 surface, but do not appear on the Ni/Ge(111)-c(2 × 8) surface. After annealing the surface above 470 K, numerous dark holes appear in the surface (Figure 4a). Interestingly, some of them are housing rather unusual objects: triangular islands which contain triangular-shaped protrusions in each apex. We refer to them as triple-holes and speculate that they contain Ni. After annealing the surface above 670 K, large islands with elongated shapes (hereafter long islands) develop in coexistence with the triple-holes. Some long islands are enclosed by circles in the large-scale image in Figure 4b, and an example island is zoomed in the left part of Figure 4c. It is seen that the edges of the long islands are aligned in three different directions, i.e., [-101], [1–10], and [01–1], indicated in the schematic diagram of the approved structural model of the Ag/Ge(111)-√3 × √3 surface (Figure 4c, lower right part).

Figure 5 shows structures which are commonly observed on the Ge(111)-c(2 × 8) and Ag/Ge(111)-√3 × √3 surfaces. One group includes three-dimensional hexagonal-shaped islands with no distinct pattern at their tops (Figure 5a,b). The other group contains islands with a 7 × 7 pattern (hereafter 7 × 7 islands) and somewhat triangular shape (Figure 5c,d).

**Figure 6:**
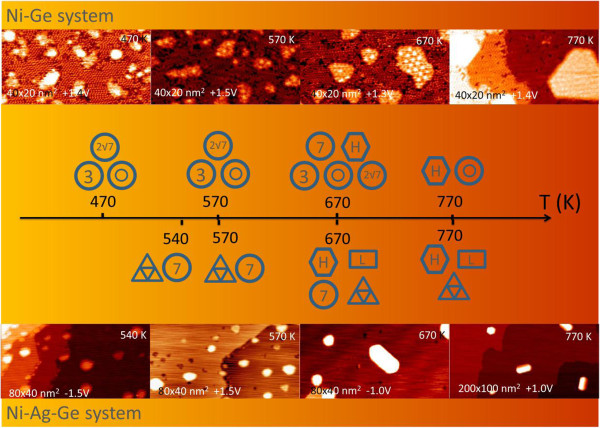
**Phase diagram for Ni/Ge(111)-c(2 × 8) and Ni/Ag/Ge(111)-√3 × √3 along with corresponding STM images.** The notations for the structural phases are indicated in Figures 3,4,5.

Figure 6 summarizes STM images of the Ni/Ge(111)-c(2 × 8) (top of Figure 6) and Ag/Ge(111)-√3 × √3 surfaces annealed within the range from 470 to 770 K (bottom of Figure 6). The hexagonal-shaped islands and those with the 7 × 7 reconstruction are common, but the others are typical of individual surfaces: ring-like structures, the 2√7 × 2√7 islands, the 3 × 3 on the Ni/Ge(111)-c(2 × 8) vs. triple-holes and long islands on the Ag/Ge(111)-√3 × √3. A brief description of the individual structures is presented above. The notations for the structural phases are indicated in Figures 3,4,5. Below, we encapsulate our observations in terms of the thermal evolution of the surfaces:

1. *Ni/Ge(111)-c(2 × 8) surface.* Even at RT, deposited Ni atoms react with the substrate forming Ni-containing clusters. When the temperature reaches 470 K, the reaction proceeds to create Ni-containing islands with the 2√7 × 2√7 and 3 × 3 reconstructions as well as the ring-like defects. At 670 K, in addition to the latter structures, the hexagonal and 7 × 7 islands appear here and there within the c(2 × 8) matrix. An increase in temperature causes the hexagonal islands to grow in size at the expense of all other types of islands. Finally, at 770 K, only the hexagonal islands remain on the surface. In the inter-island area, the ring-like features are clearly resolved.

2. *Ni/Ag/Ge(111)-√3 × √3 surface*. At RT, Ni nucleation is determined by the formation of clusters. At around 540 K, the triple-holes and the 7 × 7 islands commence forming. The latter are also observed to form on the Ni/Ge(111)-c(2 × 8) surface but at a higher temperature. After annealing at 670 K, the hexagonal and the long islands form in coexistence with all above-mentioned structures. It is likely that the clusters which were initially trapped in the triple-holes develop into regular islands upon annealing. The islands grow in size with the increase in temperature at the cost of 7 × 7 islands. Finally, at 770 K, the hexagonal and long islands coexist with the triple-holes.

The formation of defects, differing in appearance (i.e., the ring-like defects on the Ge(111)-c(2 × 8) surface vs. the triple-hole defects on the Ag/Ge(111)-√3 × √3 surface), indicates that the mixing between Ni and Ge proceeds on both surfaces through different mechanisms. Generally, however, the presence of 1 ML Ag on the Ge(111) surface retards the inter-diffusion between Ni adatoms and Ge substrates, at least at temperatures below 670 K. This is why the formation of the Ni-containing 2√7 × 2√7 and the 3 × 3 islands is prevented on the Ag/Ge(111)-√3 × √3 surface.

By analyzing a number of images taken after annealing at the final temperature, we have found that the total volume of islands is several times greater than the volume which should be expected from the amount of deposited Ni. This means that Ni reacts with Ge atoms to form Ni-containing islands, perhaps the long islands and/or the hexagonal islands.

The formation of the long islands indicates that the Ag/Ge (111)-√3 × √3 surfaces provide Ni, Ge, and Ni_*x*_Ge_*y *_clusters with a lower surface diffusion energy. As a result, the formation of the long islands takes place only on the Ge(111) surface with an Ag buffer layer.

## Conclusions

We have presented the STM results about Ni-containing nano-sized islands, as obtained on the Ge(111)-c(2 × 8) and Ag/Ge(111)-√3 × √3 surfaces after Ni deposition and annealing within the range from 470 to 770 K. On both surfaces, the appearance of defects which are typical of the whole range of annealing temperature has been observed. Apart from some types of islands, which appear on the individual surfaces, the formation of some structures common for both studied surfaces has been recorded. We argue that the Ag layer prevents deposited Ni atoms from reacting with the Ge surfaces, at least at temperatures below 670 K. At a higher temperature, however, the formation of Ni-containing islands must be assumed in order to account for the formation of islands with a large total volume as well as the appearance of structures that are also observed on the Ni/Ge(111)-c(2 × 8) surface.

## Abbreviations

RT: Room temperature; STM: Scanning tunneling microscopy; TMs: Transition metals.

## Competing interests

The authors declare that they have no competing interests.

## Authors’ contributions

T-YF conceived of the study and wrote the manuscript. AT was involved in carrying out the experiment and drafting the manuscript. X-LH and J-HL were involved in carrying out the experiment. P-IH and M-KJ analyzed the data. All authors read and approved the final version of the manuscript.
